# Infant Movement Classification Using Pose-Based Motion Features and a Learned Hybrid k-Nearest Neighbor Model

**DOI:** 10.3390/bioengineering13101200

**Published:** 2026-10-15

**Authors:** Ali Ari, Ecem Yıldız Çangur, Kamile Uzun Akkaya, Pelin Atalan Efkere, Berna Gurler Ari, Bülent Elbasan, Abdulkadir Sengur, Yan Tian

**Affiliations:** 1 Department of Computer Engineering, Faculty of Technology, Gazi University, Ankara 06654, Turkey; 2 Department of Physical Therapy and Rehabilitation, Faculty of Health Sciences, Gazi University, Ankara 06654, Turkeybulentelbasan@gazi.edu.tr (B.E.); 3 Department of Computer Engineering, Engineering Faculty, Turkish National Defence University, Ankara 06420, Turkey; 4 Electrical and Electronics Engineering, Faculty of Technology, Fırat University, Elazığ 23100, Turkey; 5 School of Computer Science, Zhejiang Gongshang University, Hangzhou 310018, China

**Keywords:** infant movement classification, pose estimation, learned hybrid kNN

## Abstract

**Background/Objectives:** Objective assessment of infant movement remains challenging because clinical assessments mostly depend on expert visual inspection and subjective scoring. This study introduces a pose-based movement analysis framework that converts raw RGB infant videos into resilient spatiotemporal representations and executes multi-class infant movement classification utilizing a lightweight learned hybrid k-nearest neighbor (kNN) model. **Methods:** The proposed pipeline extracts 2D body keypoints from each video frame, applies shoulder-centered spatial normalization to reduce scale and translation variability, and divides recordings into overlapping temporal windows. Within each segment, a small set of interpretable kinematic descriptors captures joint speed statistics, bilateral symmetry, inter-limb coordination, postural distances, movement smoothness, directional variability, entropy, and ratio-based relational features. This results in a fixed 60-dimensional feature vector for each segment. The pose-derived features were evaluated using a learned hybrid kNN classifier with adaptive similarity weighting and conventional classifiers under the same subject-wise evaluation protocol. The framework was evaluated using both segment-wise stratified 5-fold cross-validation and subject-wise grouped 5-fold cross-validation. **Results:** Segment-wise CV yielded an accuracy of 96.44%, while subject-wise grouped CV achieved 94.80%, indicating strong performance under both evaluation settings. **Conclusions:** The study contributes a pose-based framework and the BERA-GAZI-RGB dataset for infant movement classification. Learned Hybrid kNN performed similarly to conventional kNN, without demonstrating a consistent advantage of hybrid weighting. Further validation is needed to assess its potential for early movement screening.

## 1. Introduction

Movement is inherent in human nature from the fetal period and is considered an indicator of the healthy functioning of higher centers. Spontaneous movements are simple or complex, involuntary movements produced by the immature nervous system that occur without the need for any sensory input. Spontaneous movements are thought to be triggered by central pattern generators (CPGs) located in the spinal cord and brainstem. Sensory stimuli from the periphery and pathways descending from cortical and subcortical structures influence the modulation of CPGs. In the first year of life, infants exhibit a rich repertoire of spontaneous and voluntary movements that rapidly develop in terms of complexity, variability, and coordination. These early movement patterns are not only a prerequisite for later motor skills but also provide a critical understanding of neural maturation and organization. Atypical neuromotor development in infancy is frequently associated with a range of neurodevelopmental disorders, including cerebral palsy, developmental delay, and autism spectrum disorder. More importantly, deviations from spontaneous movement patterns may emerge several months before the onset of pronounced clinical symptoms, highlighting the importance of early detection and intervention [1]. Movement-analysis framework allows for timely neurodevelopmental interventions that benefit neurological development. Clinical assessment of infant neuromotor development traditionally relies on standardized observational tools such as the General Movement Assessment (GMA) and structured developmental scales, including the Bayley Scales of Infant and Toddler Development. Among these, the GMA has been widely validated as a sensitive and non-invasive method for identifying infants at high risk of neurological disorders [2]. However, despite its clinical value, the GMA requires comprehensive testing.

Recent advances in machine learning (ML), computer vision, and artificial intelligence have brought new possibilities for the objective and automated analysis of infant movements. ML-based systems can reveal complex spatio-temporal features from high-dimensional data such as video recordings and detect subtle details associated with neuromotor dysfunction. In this way, they offer the potential for more objective analysis, improved scalability, and support for continuous monitoring of infant development in both clinical and home settings [3,4].

Spontaneous movements observed in early infancy are widely accepted as indicators of neurological health. General movements (GMs), which emerge in fetal life and continue until approximately 20 weeks after birth, involve complex, variable, and fluid whole-body movement patterns [5,6]. While these movements exhibit a rich variety and adaptability in typically developing infants, infants with neurological disorders often exhibit monotonous, rigid, or poorly coordinated movements [7,8]. The General Movement Assessment (GMA), developed by Prechtl and colleagues, is considered one of the most reliable tools for early neurological assessment. Numerous studies have shown that GMA has high predictive validity for cerebral palsy and other neurodevelopmental disorders [2,9]. Despite these strengths, GMA requires specialized training, and its application is limited by subjectivity, evaluator fatigue, and the limited availability of specialist clinicians.

To overcome the limitations of manual assessment, many studies have explored automated approaches for analyzing infant movements. Adde et al. [10] conducted a study on computer-based video analysis to enable the early detection of cerebral palsy. In the method proposed by the authors, motion images were created based on the differences between consecutive frames in video clips. Using these images, the spatial and temporal variability of the amount of movement and its centre was calculated as quantitative parameters. Consequently, it has been demonstrated that variability in the centre of motion provides 85% sensitivity and 71% specificity in predicting the risk of CP development. However, the lack of a deep learning-based method limited the automatic learning of complex motor skills and subtle temporal variations. While these methods proved their applicability, they were sensitive to environmental factors such as lighting changes, camera movement, and obscuration. Ihlen et al. [11] proposed a new machine learning model called Computer-Based Infant Movement Assessment (CIMA) that can be used in the clinic for early CP prediction based on video recordings of infants. The model is designed to evaluate movement pattern ratios associated with CP risk using the time–frequency decomposition of all the infant’s body movement trajectories. The results showed that the CIMA model, with a sensitivity of 92.7% and a specificity of 81.6%, could be an alternative to observational GMA in clinical CP prediction.

Orlandi et al. [12] proposed a model using a dataset of retrospective videos with clinically analysed GMA results, incorporating a skin model for segmentation and large displacement optical flow (LDOF) for movement tracking. The model was evaluated according to suitability criteria for automated analysis. WEKA was used to classify movements as typical or atypical. An accuracy of up to 92% was achieved in predicting cerebral palsy early. It was stated that a computer-based assessment could be used by clinicians as an objective alternative tool for the early diagnosis of cerebral palsy and could improve functional outcomes by facilitating early intervention. While these methods increased robustness, they often required manual annotations or manually defined thresholds, limiting their scalability and generalizability. McCay et al. [13] proposed a body-part-based ensemble framework integrated with a pose-based feature fusion model to improve the accuracy of CP detection in infants. The authors used the MINI-RGBD and RVI-38 datasets. The MINI-RGBD dataset consists of 12 infants’ video recordings obtained in the hospital over a period of 6 months. The RVI-38 dataset consists of video recordings of 38 infants between three and five months post-term. As a result, the proposed framework achieved a high accuracy rate of 98.4%. This demonstrates the potential of the framework to improve the accuracy of cerebral palsy diagnosis. However, the limitations of the study include the limited sample size, the absence of depth and 3D motion information (only 2D pose estimation), and the fact that it has not yet been tested in multicentre settings or under different camera conditions.

Yang et al. [14] aimed to preserve ecological validity by utilizing a dataset consisting of videos recorded by parents in the home environments of 90 infants who had been discharged from an early evaluation and intervention center in Taiwan, following standardized recording guidelines. The authors proposed four pose recognition models, including ViTPose, HRNet, DARK, and UDP. Four different machine learning classification models, including random forest, support vector machine, logistic regression, and XGBoost, were used to predict children’s developmental status. They obtained the highest performance with a 94% accuracy rate using the random forest model. Moccia et al. [15] proposed a framework for estimating limb position in premature infants from depth images using spatio-temporal features. Limb position estimation was performed with a deep learning framework that incorporated detection and regression convolutional neural networks (CNNs) for gross and fine joint localisation, respectively. The CNNs encoded temporal connections via 3D convolution. The authors used the babyPose dataset, which consists of sixteen depth videos obtained from sixteen premature infants in real clinical practice. The results demonstrated that spatio-temporal features successfully improved pose estimation performance compared with 2D models based on single-frame (spatial only) information. It was shown that deep learning approaches offer higher accuracy than conventional machine learning methods.

Ali et al. [16] proposed an interpretable and automated system based on a 2D and 3D pose estimation method to assist in the early diagnosis of cerebral palsy, as an alternative to the subjective and clinician-dependent nature of the General Movement Assessment. The authors used the MINI-RGBD and RVI-38 datasets in their study. They obtained accuracy rates of 92% and 97.37% using the MINI-RGBD and RVI-38 datasets, respectively. The small sample size of the RVI-38 dataset, the lack of access to raw videos, the synthetic nature of the MINI-RGBD dataset, and the absence of automatic learning capabilities compared with deep learning models were identified as limitations of the study.

Dalal and Triggs [17] evaluated different methods for human detection and proposed the Histogram of Oriented Gradients (HOG)-based method. Linear Support Vector Machines (Linear SVM) were used to classify the extracted HOG features. The authors used the MIT Pedestrian Dataset and the INRIA Person Dataset. The MIT Pedestrian Dataset consists of data with a more limited variety of poses and front and back views, whereas the INRIA Person Dataset consists of more challenging data with diverse poses and backgrounds. As a result, almost perfect discrimination was achieved in the MIT dataset, while a substantial improvement in positive error was observed in the INRIA dataset. The most significant limitation of the method is that it does not explicitly model the articulated structure of the human body.

In another study, Xia et al. [18] proposed a histogram-based method using 3D joint (skeleton) data for human movement recognition. Histogram of 3D Joint Positions (HOJ3D) aims to provide an independent perspective on human action recognition. The authors used the depth sensor-based MSR Action3D Dataset. As a result, they obtained an accuracy of 85–95%. Despite this level of accuracy, the method has notable limitations, as it can only be applied in environments with depth sensors and is directly dependent on the accurate extraction of skeletal data. From the perspective of infant movement analysis, histogram-based approaches have yielded promising results in capturing the temporal coordination and variability of movements. When integrated into pose-based data, histogram features provide an informative description of neuromotor behavior.

Hesse et al. [19] proposed a learned infant body model from RGB-D data to better analyze the baby’s overall body movements. The method is based on learning joint relationships with a kinematic body model specific to infant anatomy through the simultaneous estimation of 3D body points obtained from RGB and depth data. The authors used a dataset consisting of images recorded with RGB-D sensors in a laboratory setting. The study focused on pose estimation accuracy rather than classification accuracy. The results demonstrated that the proposed approach achieved approximately 90% accuracy across most joint locations.

Valdivia et al. [20] proposed a classification method using Recurrent Neural Networks to address this issue. The proposed method for classifying hemiplegia and diplegia enables the capture of complex temporal dynamics in force data through the use of long short-term memory (LSTM), gated recurrent units (GRU), bidirectional long short-term memory (BiLSTM), bidirectional gated recurrent units (BiGRU), and autoregressive integrated moving average (ARIMA) models. The results demonstrated that the proposed models were effective in capturing temporal dynamics. The highest classification accuracy (76.43%) was obtained using the BiGRU and LSTM models.

Ari et al. [21] proposed an automated infant movement classification framework combining Pose-LBP histograms with joint-specific motion ratio features extracted from RGB videos. The resulting 289-dimensional representation was classified using a cost-sensitive subspace kNN ensemble. The method achieved a reported accuracy of 99.16% under segment-wise ten-fold cross-validation. However, this evaluation does not establish generalization to unseen infants, highlighting the importance of subject-wise grouped validation.

Pellano et al. [22] investigated the explanations generated by CAM and Grad-CAM for a previously developed GCN-based cerebral palsy prediction model. They applied velocity and angular perturbations to selected body keypoints to examine their influence on predicted risk. Velocity-related changes had a greater influence than angular changes, while CAM and Grad-CAM showed partial convergence in their explanations.

Baker et al. [23] expanded this research area by investigating the usability of cradleside cameras routinely installed in neonatal intensive care units (NICUs) for deep learning-assisted general movement assessment. Unlike previous studies relying on controlled recording environments or specialized equipment, this study focused on leveraging the existing cradleside video infrastructure already used for infant monitoring and parental involvement. The authors reviewed the existing literature to evaluate whether video data captured from cot-side edge cameras are suitable for automated GMA and whether deep learning techniques can reliably analyze spontaneous infant movements in this context.

In this paper, a novel technique was developed for automated movement analysis of the infants’ videos for abnormality detection. To this end, we developed a system that integrates computer vision and advanced machine learning algorithms to objectively evaluate the processes of infant neuromotor development. The framework is built using skeletal coordinate data from raw video recordings that have been processed by deep learning-driven object detection and key point localization systems. These temporal data were divided into 4 s windows using a stride of 0.5 s, resulting in an effective overlap of 3.5 s between consecutive segments. From these, 60 different biomechanical and statistical characteristics are extracted. The feature set quantitatively characterizes infant movement using limb speed, bilateral symmetry, jerk indices, postural distances, and signal entropy. We put forward a new type of classifier called the Learned Hybrid KNN. This model goes beyond traditional methods by combining quantum-inspired similarity measures, specifically fidelity-based quantum weights, with Euclidean-based Radial Basis Function (RBF) kernels.

The main contributions of this paper are

(1) A novel infant movement dataset comprising five movement classes is introduced.

(2) A novel classifier, termed Learned Hybrid kNN, is introduced for infant movement classification.

## 2. Materials and Methods

**Figure 1:**
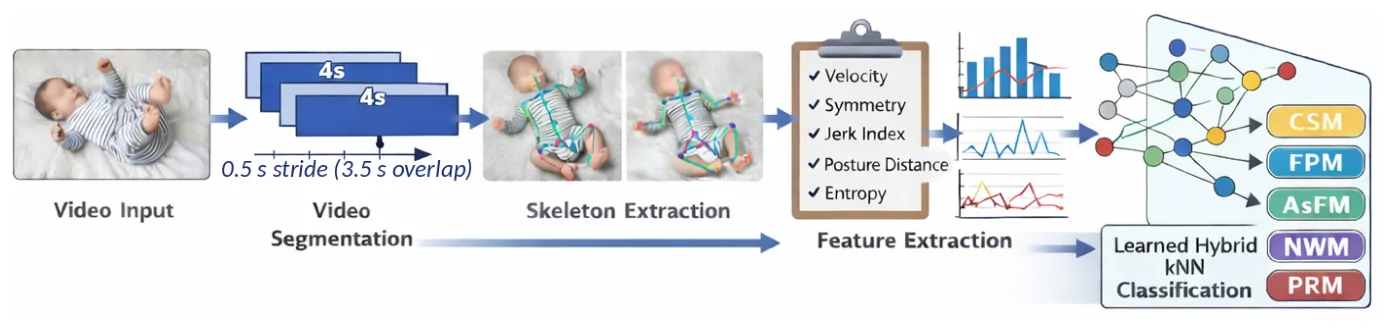
Illustration of the proposed model.

The present study proposes an automatic movement analysis system that incorporates computer vision with state-of-the-art machine learning techniques to objectively assess the processes of infant neuromotor development. Figure 1 illustrates the proposed method system. This system is based on the extracted skeletal coordinate data obtained using raw video streams with the help of deep learning-based object detection and keypoint localization architectures. The extracted pose sequences were divided into 4 s temporal windows using a stride of 0.5 s, corresponding to an effective overlap of 3.5 s between consecutive segments. Then, a set of 60 features is extracted using the segmented data, which quantifies the movements of the infants by measuring the key aspects of the movements, such as the speed of the limbs, the extent of symmetry, jerk, distance, and entropy of the posture of the infants. A new classifier named Learned Hybrid KNN is introduced, which enhances the capabilities of the conventional classifier by incorporating the Euclidean distance-based RBF kernel with the quantum-based similarity measure, i.e., fidelity-based quantum weights. During the training phase, the optimal hyperparameters for the weighting scheme used by the classifier, i.e., alpha, which corresponds to the hybrid ratio, sigma, which corresponds to the RBF width, and *p*, which corresponds to the power of the quantum weights, are obtained using the grid search method for each fold of the dataset used for training the classifier. The performance of the proposed system is tested using the cross-validation method for evaluating the applicability of the system to different scenarios, i.e., for evaluating the generalization ability of the system for the purpose of classifying the motor movements with high precision using the proposed Learned Hybrid KNN classifier by determining the weights of the neighbors using the distance vector for each test instance. The proposed method provides a quantitative framework for infant movement analysis and may serve as a screening-support approach after further prospective and longitudinal clinical validation.

### 2.1. Proposed Framework Overview

Let an RGB video be represented as a temporal sequence of frames $V=\left\{I_{1},\;I_{2},\ldots ,I_{N_{f}}\right\}$, where *I_n_* denotes the frame sampled at time *t_n_* = *n*Δ*t* and Δ*t* shows the temporal sampling interval. Using pose-based motion analysis, the proposed framework turns raw RGB videos into fixed-length numerical representations. The pipeline includes temporal sampling, person detection, pose estimation, pose normalization, overlapping temporal segmentation, and segment-level feature extraction. Every video is broken up into several overlapping segments, and a fixed-dimensional feature vector that works for machine learning-based classification describes each segment.

### 2.2. Pose Estimation and Normalization

For each sampled frame *I_n_*, a person detection model is applied to identify candidate human regions in the given frame. Let $B_{n}=\left\{B_{n}^{1},\ldots ,B_{n}^{K}\right\}$ denote the set of detected bounding boxes with associated confidence scores. The bounding box with the maximum confidence score is selected.

Person detection was performed using RTMDet-Tiny, and detections with confidence scores below 0.25 were discarded. Top-down pose estimation was performed using HRNet-W32, and key points with confidence scores below 0.20 were treated as invalid.

Bn=argmaxBnk∈BnscoreBnk

If no person is detected, the frame pose representation is defined as a zero vector. Given the selected bounding box *B_n_*, a pose estimation model extracts *J* = 17 body joints. The 2D image coordinate of joint *j* at frame *n* is given by

*p_j_*(*n*) = [*x_j_*(*n*), *y_j_*(*n*)]

with a corresponding validity indicator

*υ_j_*(*n*) ∈ {0,1}.

To remove translation and scale variations between videos, a body-centered normalization scheme is applied. The left and right shoulder joints are denoted by indices *L_S_* and *R_S_*. The pose center is defined as

$$c\left(n\right)=\frac{p_{L_{S}}\left(n\right)+p_{R_{S}}\left(n\right)}{2}$$

The scale factor is defined as the Euclidean distance between the shoulder joints:

$$s\left(n\right)=\left\|p_{L_{S}}\left(n\right)-p_{R_{S}}\left(n\right)\right\|$$

Each valid joint is normalized as

$${\tilde{p}}_{j}=\frac{p_{j}\left(n\right)-c\left(n\right)}{s\left(n\right)}$$

If a joint is invalid or if *s*(*n*) = 0, the normalized coordinate is set to zero. All normalized joint coordinates are concatenated to form a fixed-length frame-level pose vector:

$$s\left(n\right)\;=\left[{\tilde{x}}_{1}\left(n\right),\;{\tilde{y}}_{1}\left(n\right),\ldots ,{\tilde{x}}_{17}\left(n\right),{\tilde{y}}_{17}\left(n\right)\right]\in R^{34}$$

This representation ensures consistency across frames and videos.

### 2.3. Temporal Segmentation and Feature Extraction

The normalized frame vectors *s*(*n*) are stored in a temporal buffer. Fixed-length temporal segments are constructed using a sliding window approach. Let the segment duration be *L* = 4 s. The number of frames per segment is

$$T=\left\lfloor \frac{L}{\Delta t}\right\rfloor$$

The normalized frame vectors *s*(*n*) were stored in a temporal buffer and divided into fixed-length segments using a sliding-window procedure. Let *L* = 4 s denote the segment duration and Δ*t* = 0.05 s the temporal sampling interval. Accordingly, the number of frames in each segment was calculated as

$$T=\mathrm{floor}\left(\frac{L}{\Delta t}\right)=80$$

The stride between consecutive segments was *H* = 0.5 s, corresponding to a frame shift of

$$h=\mathrm{floor}\left(\frac{H}{\Delta t}\right)=10\;\mathrm{frames}.$$

Therefore, consecutive segments shared an effective overlap of *L* − *H* = 3.5 s, corresponding to 70 frames. The starting frame of segment *m* was defined as

*n_m_* = 1 + (*m* − 1)*h*,

and the corresponding segment was constructed as

*S_m_* = {*s*(*n_m_*),*s*(*n_m_* + 1), …, *s*(*n_m_* + *T* − 1)}.

For a video containing *N_f_* sampled frames, the number of complete candidate segments was calculated as

*N_v_* = *floor*((*N_f_* − *T*)/*h*) + 1.

Equivalently, for a video duration of *D* seconds, the number of candidate segments was *floor*((*D* − *L*)/*H*) + 1. Thus, a 20 s video produced 33 candidate segments before pose-quality screening. For a given segment *S_k_* the pose sequence is reshaped into a tensor

*P* ∈ ℝ*^T^*^×17×2^

Across the 400 standardized 20 s recordings, this procedure generated 13,200 candidate windows (33 windows per recording). Pose quality was assessed for each window using the proportion of valid joint-frame observations. Each 4 s window contained 80 frames and 17 joints, yielding 1360 joint-frame observations. According to the implemented preprocessing code, a joint-frame observation was counted as valid when at least one normalized coordinate was non-zero. The validity ratio was calculated across all 1360 observations, and windows with a validity ratio below 0.55 (fewer than 748 valid joint-frame observations) were excluded. The 400 recordings generated 13,200 candidate windows. The abnormal-fidgety category comprised two recordings and 66 candidate windows; this category was excluded from the five-class analysis because it contained only one infant. Among the remaining 13,134 candidate windows, 7540 were excluded during preprocessing and 5594 were retained for feature extraction and classification. Thus, the overall difference of 7606 between generated and retained windows includes both the 66 windows removed with the excluded category and the 7540 windows excluded from the five analytical classes.

Joint velocity is computed as the temporal difference in joint positions:

*v_j_*(*n*) = *p_j_*(*n* + 1) − *p_j_*(*n*)

Joint speed is defined as the magnitude of velocity:

*u_j_*(*n*) = ‖*v_j_*(*n*)‖

Upper-limb and lower-limb motion signals are computed by averaging speeds of the corresponding joints. Bilateral symmetry is measured as the absolute difference between left and right limb speeds. For each motion signal *x*(*n*) statistical descriptors are computed, including mean, standard deviation, and root-mean-square:

$$\mu_{x}=\frac{1}{N}\sum_{n}x\left(n\right),\;\sigma_{x}=\sqrt{\frac{1}{N}\sum_{n}{\left(x\left(n\right)-\mu_{x}\right)}^{2}},\;{\mathrm{RMS}}_{x}=\sqrt{\frac{1}{N}\sum_{n}x\left(n^{2}\right)}$$

Temporal coordination between paired signals is modeled using normalized cross-correlation:

$$\rho_{\mathrm{ab}}\left(\tau \right)=\frac{\sum_{n}a\left(n\right)b\left(n+\tau \right)}{\sqrt{\sum_{n}a{\left(n\right)}^{2}\sum_{n}b{\left(n\right)}^{2}}}$$

from which the maximum correlation coefficient and corresponding time lag are retained.

Postural characteristics are captured using distances between anatomical landmarks, such as shoulder width and hip width, as well as distances of distal joints to body midpoints. Movement smoothness is characterized using jerk signals defined as temporal differences in speed:

*jerk*(*n*) = |*u*(*n* + 1) − *u*(*n*)|

Additional descriptors include direction-change counts, entropy-based measures derived from speed histograms, and ratio-based features that capture relative motion and posture patterns.

All descriptors are concatenated into a single fixed-length feature vector

*f_k_* ∈ ℝ^60^

This fixed-dimensional representation enables consistent comparison across segments and videos.

### 2.4. k-Nearest Neighbors (KNN)

Let us consider a training dataset, which is defined as (*x_i_*_,_ *y_i_*) pairs,

$$D={\left\{\left(x_{i,\;}y_{i}\right)\right\}}_{i=1}^{N}$$

where *x_i_* ∈ ℝ*^d^* denotes the feature vector of the *i*^th^ sample and *y_i_* ∈ {1,2, …, *C*} denotes its class label. For a test sample *x* the distance between *x* and a training sample *x_i_* is calculated using the Euclidean distance,

$$d\left(x,x_{i}\right)={\left\|x-x_{i}\right\|}_{2}$$

The set of the *k* nearest neighbors of *x* is defined as

Nkx=argminkdx,xi

The predicted class label is obtained by majority voting among the nearest neighbors:

y^=argmaxc∈1,…,C∑xi,yi∈NkxIyi=c

where 𝕀(.) is the indicator function.

### 2.5. Learned Hybrid k-Nearest Neighbors

The proposed Learned Hybrid KNN extends the classical KNN by introducing a learnable distance weighting mechanism that combines classical distance-based similarity with a quantum-inspired similarity measure. Given a test sample *x*, neighbors are selected using the Euclidean distance as in standard KNN. Let *N_k_*(*x*) denote the set of nearest neighbors. For each neighbor *x_i_* ∈ *N_k_*(*x*), two complementary similarity measures are computed,

$$s_{\mathrm{RBF}}\left(x,x_{i}\right)=\exp\left(-\frac{{\left\|x-x_{i}\right\|}_{2}^{2}}{\sigma^{2}}\right)$$

where *σ* > 0 controls the width of the radial basis function. After 𝓁_2_-normalization of feature vectors,

$$\tilde{x}=\frac{x}{{\left\|x\right\|}_{2}},\;\tilde{x_{i}}=\frac{x_{i}}{{\left\|x_{i}\right\|}_{2}}$$

the quantum-inspired similarity is defined as

$$s_{Q}\left(x,x_{i}\right)={\left|{\tilde{x}}^{T}{\tilde{x}}_{i}\right|}^{2}$$

The final hybrid similarity weight is computed as a convex combination:

$$w\left(x,x_{i}\right)=as_{\mathrm{RBF}}\left(x,x_{i}\right)+\left(1-a\right)s_{Q}{\left(x,x_{i}\right)}^{p}$$

where *a* ∈ [0,1] balances the contribution of classical and quantum-inspired similarities, and *p* ≥ 1 controls the nonlinearity of the quantum-inspired term. The predicted class label is obtained by weighted voting:

y^=argmaxc∈1,…,C∑xi,yi∈Nkxwx,xiIyi=c

Hyperparameter selection was performed separately within each outer training fold through nested grouped cross-validation. In both the inner and outer validation loops, all recordings and overlapping segments obtained from the same infant were kept within the same fold. Candidate hyperparameter configurations were evaluated using only the subjects included in the corresponding outer training partition. The grid search considered the hybrid weighting coefficient α within the range 0 ≤ α ≤ 1, the quantum-weighting power *p* ∈ {1, 2, 3, 4}, the RBF width *σ* ∈ {0.30, 0.60, 1.00, 1.50, 2.50, 4.00}, and the number of nearest neighbors *k* ∈ {1, 3, 5, 7}. The configuration achieving the highest mean macro-averaged F1-score across the inner folds was selected. The model was then retrained using the complete outer training partition and evaluated once on the held-out outer test subjects. Feature-standardization parameters were also estimated exclusively from the relevant training partition and subsequently applied to the corresponding validation or test data. Thus, no information from the outer test subjects was used during preprocessing, model selection, or hyperparameter optimization. When α = 1, the method reduces to classical RBF-weighted kNN, whereas α = 0 corresponds to a purely quantum-inspired similarity-weighted classifier.

## 3. Experimental Works and Results

### 3.1. Dataset

This study introduced the BERA-GAZI-RGB dataset, which was collected at the Developmental Physiotherapy and Pediatric Rehabilitation Unit of the Faculty of Health Sciences, Gazi University, between 14 January and 14 December 2025. The dataset comprised 400 standardized 20 s RGB video clips derived from recordings of 200 high-risk infants. Each infant contributed exactly two recordings, and both recordings of an infant were obtained within the same developmental assessment period. The two recordings of an infant were therefore assigned to the same GMA category and could not receive different movement labels. Assessments were carried out in two post-term developmental periods defined by corrected age: the writhing period (0–9 weeks) and the fidgety period (9–20 weeks). Of the 200 infants, 77 were assessed in the writhing period and 123 in the fidgety period. The demographic, clinical, and recording characteristics of the study population are summarized in Table 1. The recordings were evaluated using the General Movement Assessment and assigned to one of five movement categories: Normal Writhing Movements (NWM), Poor Repertoire Movements (PRM), Cramped-Synchronized Movements (CSM), Fidgety Present Movements (FPM), and Absent Fidgety Movements (AsFM). NWM, PRM, and CSM represented the writhing period, whereas FPM and AsFM represented the fidgety period. Two recordings obtained from one infant were classified as abnormal fidgety movements. Because this category contained a single infant, it could not be represented in both the training and the test partitions of a subject-wise five-fold design, and it was therefore excluded from the analytical dataset. The class denoted AbFM in the previous version of the manuscript corresponds to the fidgety-present category of the clinical records and has been renamed FPM throughout to remove this inconsistency. Following temporal segmentation and preprocessing, 5594 segments were retained: 2306 FPM segments (41.22%), 911 AsFM segments (16.29%), 331 CSM segments (5.92%), 663 NWM segments (11.85%), and 1383 PRM segments (24.72%).

Sex was documented for 64 of the 200 infants, gestational age for 45, and clinical risk-factor entries for 44; these variables are reported in Table 1 together with their denominators. Exact corrected age at the level of the individual recording could not be recovered from the available records; therefore, class-specific corrected-age distributions cannot be reported. The age information in Table 1 describes developmental-period ranges, while Table 2 reports class-by-period composition rather than class-specific age distributions. Because the demographic variables were documented for subsets of the cohort, class-stratified demographic summaries are not reported.

All video recordings were assessed using the General Movements Assessment by a single pediatric physiotherapist with 20 years of professional experience and GMA training certification. The two recordings of each infant were reviewed within the same developmental assessment period and categorized according to the standard GMA categories. The assessor was blinded to the infants’ clinical diagnoses during the GMA assessments. No independent second ratings or adjudication assessments were available, and an inter-rater agreement statistic such as weighted kappa cannot be computed. The absence of independent ratings limits the assessment of reference-label reliability. Future data collection should include independent blinded ratings and adjudication of disagreements.

The composition of the dataset at the infant, recording, and segment levels is reported in Table 2. Because every recording was standardized to 20 s, each recording produced 33 candidate windows, so the class-level candidate, excluded, and retained window counts follow directly from the recording counts and the retained segment counts. They sum to the 13,200 candidate windows, the 7606 windows that were not retained, and the 5594 retained segments reported in Section 2.3. Window retention differed between classes, ranging from 25.8% for NWM to 83.6% for CSM, which indicates that pose-quality screening did not act uniformly across categories. This class-dependent retention is discussed as a potential source of bias in Section 4.

### 3.2. Results

The preprocessing, 60-feature extraction, and evaluation pipeline was implemented in Python 3.11.16 on a computer equipped with an NVIDIA GeForce RTX 3090 GPU and 24 GB of RAM. We used fixed-length overlapping windows to divide the time into segments. Each segment had a duration of 4 s and contained 80 frames sampled at 0.05 s intervals. Consecutive segments were generated using a stride of 0.5 s, corresponding to a shift of 10 frames and an effective overlap of 3.5 s. This resulted in 80 sampled frames per segment. A two-stage pose extraction pipeline was used for each frame that was sampled. First, a person detector was used to identify the infant region. If more than one person was detected, the bounding box with the highest confidence score was chosen. Second, an HRNet-based key point detector was used to find 2D human skeleton key points inside this bounding box. This gave us 17 body joints per frame. If no valid person or key points were found, the frame was represented by a zero vector to keep the length of the time sequence the same. The detected 2D joint coordinates were normalized to reduce translation and scale variability. The skeleton was centered between the left and right shoulders and scaled by the inter-shoulder distance. The normalized pose was then shown as a 34-dimensional vector for each frame, with the *x* and *y* coordinates of 17 joints and invalid joints set to zero. A fixed-length feature vector was constructed for each segment by taking motion and posture descriptors from the normalized key point trajectories. We calculated joint velocities over time and turned them into speed features. We developed separate features for the wrists, ankles, upper-body joints, and lower-body joints. From these features, various descriptors such as the mean, standard deviation, and root mean square; symmetry measures of the left and right limbs; synchronization cues such as maximum correlation and lag of specific motion signals such as posture-based distances such as shoulder width, hip width, and limb-to-torso distances; jerk-based cues indicating abrupt changes in motion; direction change counts indicating how many times something is changing direction; entropy-based cues indicating the rates at which something is moving; and various ratio-based features indicating how something is changing over time. In a set order, all of these scalar descriptors were collected together in a 60-dimensional vector. The final dataset comprised 5594 segments distributed across the five GMA classes as follows: 2306 FPM segments (41.22%), 911 AsFM segments (16.29%), 331 CSM segments (5.92%), 663 NWM segments (11.85%), and 1383 PRM segments (24.72%).

Two evaluation levels are reported. The original segment-wise five-fold analyses are retained as descriptive secondary results; because overlapping windows from the same infant could occur in different folds, these results are not used as evidence of subject-level generalization. The primary evaluation used stratified, infant-wise grouped outer five-fold cross-validation, with all recordings and overlapping windows from the same infant assigned to one fold. Hyperparameter selection used grouped four-fold inner cross-validation within each outer training partition, and feature standardization was fitted on training data only. The primary model comparison and the corresponding ablation analysis followed this subject-wise grouped protocol. The baseline kNN classifier used a single nearest neighbor. This choice is based on a strict nearest-neighbor rule, which works best in high-dimensional feature spaces when there is enough training data. Euclidean distance was used to measure similarity, and feature standardization was used in each training fold to avoid bias caused by differences in feature scales. The Hybrid QDC classifier, where QDC stands for Quantum Distance Classifier, takes the idea of “nearest neighbor” and adds a quantum-inspired similarity measure to it. First, candidate neighbors are found using Euclidean distance. Then, class voting is done using a fidelity-based similarity calculated from normalized feature vectors. The number of neighbors that will help make the final decision is not fixed. Instead, it is chosen adaptively within a range based on the confidence margin between the two classes that are competing. There are two margin thresholds that control this behavior. When the decision is clear, the classifier can use fewer neighbors, and when the class separation is unclear, it can use more. A power factor that emphasizes very similar samples makes the quantum-inspired similarity stronger. The RFF + KNN classifier uses both Random Fourier Features and the nearest neighbor method to perform classification. This method uses a random projection that is meant to be like a radial basis function kernel to map the original feature vectors into a space with more dimensions. The number of dimensions in this random feature space was chosen to strike a good balance between how well the kernel approximation works and how quickly it can be done. After this change, classification is done with Euclidean distance and a single nearest neighbor. Before using the KNN classifier, the LDA metric performs Linear Discriminant Analysis as a preprocessing step. LDA moves the original features into a lower-dimensional space that makes it easier to tell the different classes apart. The number of classes automatically determines the dimensionality of this subspace, according to the standard way of doing LDA. Next, Euclidean distance and one neighbor are used to do nearest neighbor classification in the projected space. The proposed classifier for this research is the Learned Hybrid KNN method. This method keeps the simplicity of nearest neighbor classification while adding a similarity function that can be learned. Euclidean distance is used to rank neighbors, but a weighted combination of two similarity measures, classical radial basis function similarity and quantum-inspired fidelity similarity, decides how much each neighbor affects the final decision. An internal validation process in each training fold automatically chooses the right balance between these two parts and the strength of the quantum-inspired contribution. This data-driven parameter selection lets the suggested method change its similarity model to fit the dataset’s features while still being strong and not needing manual tuning.

**Figure 2:**
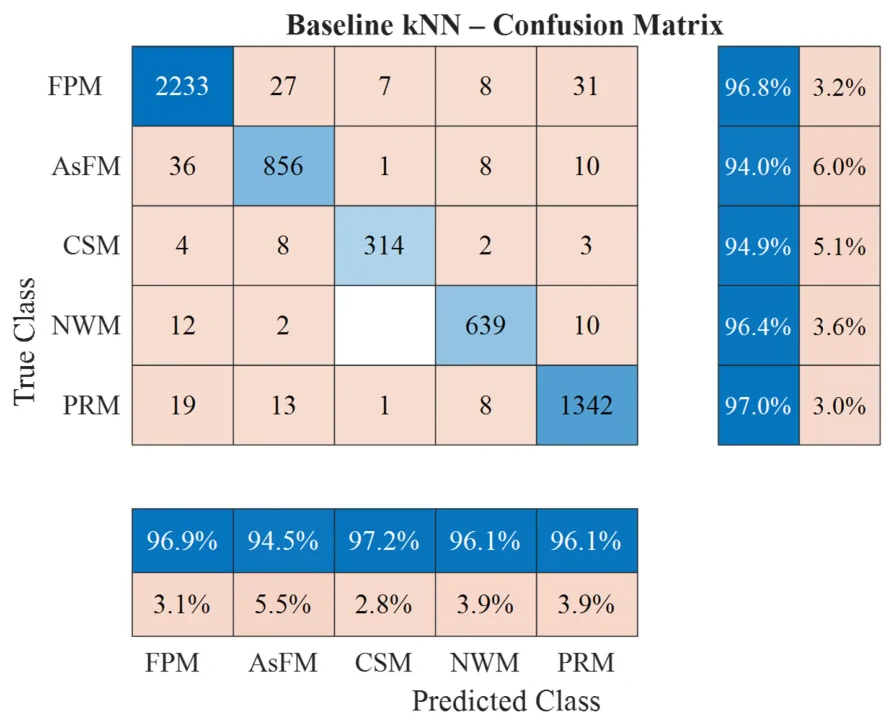
Confusion matrix of the Baseline KNN classifier under descriptive segment-wise five-fold cross-validation.

Figure 2 illustrates the confusion matrix and class-based evaluation values for the Baseline kNN method for the five movement classes: Fidgety Present Movements (FPM), Absent Fidgety Movements (AsFM), Cramped Synchronized Movements (CSM), Normal Writhing Movements (NWM), and Poor Repertoire Movements (PRM). The classifier achieved high class-wise performance. The PRM and FPM classes had the highest true positive rates, at 97.0% and 96.8%, respectively. Most of the predictions are correct and fall along the diagonal, but there are a few small mistakes. For example, 36 times AsFM was incorrectly labeled as FPM and 31 times FPM was predicted as PRM. The precision metrics at the bottom back up the model’s reliability even more, especially for the CSM class, which has a peak precision of 97.2%. The classifier’s performance is consistent overall, with accuracies for each class above 94%. This shows that the kNN baseline is a good predictor for this dataset.

Table 3 shows the performance evaluation metrics for the Baseline KNN classifier for 5-fold cross-validation. With a standard deviation of 0.0067, the average accuracy is 0.9625. The mean sensitivity value is 0.9582, with a standard deviation of 0.0057. The mean specificity is 0.9896, with a standard deviation of 0.0018. The average F1-score is 0.9599, and the standard deviation is 0.0067.

**Figure 3:**
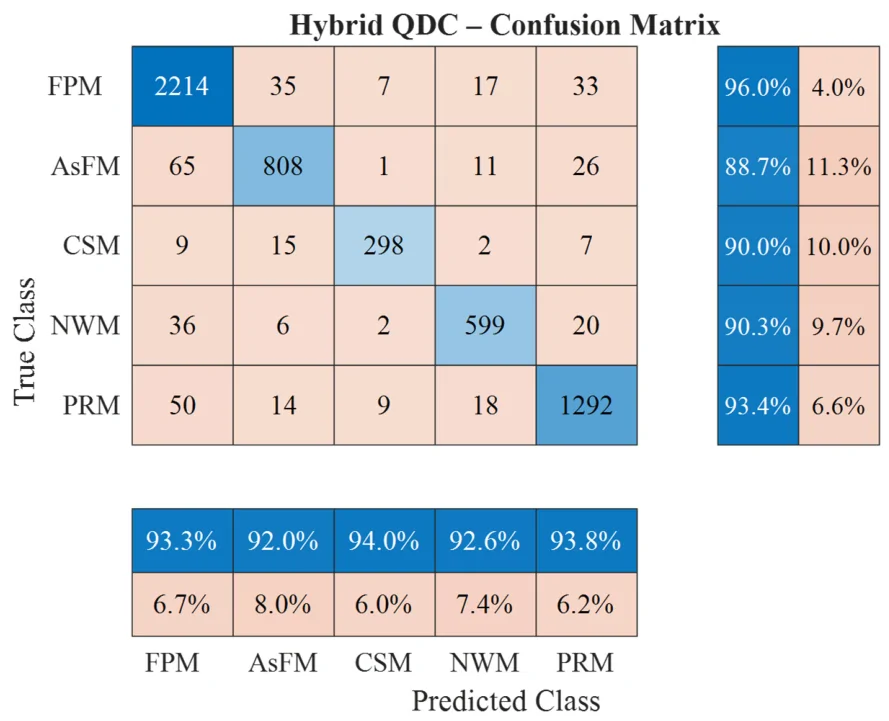
Confusion matrix of the Hybrid QDC classifier under descriptive segment-wise five-fold cross-validation.

The confusion matrix in Figure 3 shows the results of the Hybrid QDC method’s classification. It shows that the model correctly identifies 2214 samples of the FPM class, which is its highest individual sensitivity at 96.0%. The PRM class also achieved strong performance, with 1292 correct predictions and a success rate of 93.4%. However, the AsFM and CSM classes showed comparatively lower sensitivities, with true positive rates of 88.7% and 90.0%, respectively. The error distribution shows that misclassifications often happen between categories that are similar. For example, the model gets 65 AsFM samples and 50 PRM samples wrong by labeling them as FPM. Even with these overlaps, the accuracy for the predicted classes remained high, with the CSM category being the most reliable at 94.0%.

Table 4 details the Hybrid QDC classifier’s performance. Its accuracy is 0.9315, sensitivity is 0.9170, and F1-score is 0.9238 on average. That is, it balances precision and recall quite well. A particular standout feature is its specificity at an average of 0.9805; it generates very few false positives across the folds. These small standard deviations, 0.0056 for accuracy and 0.0082 for F1, suggest that the classifier is insensitive to training samples in each fold, demonstrating statistical stability that is fairly solid.

**Figure 4:**
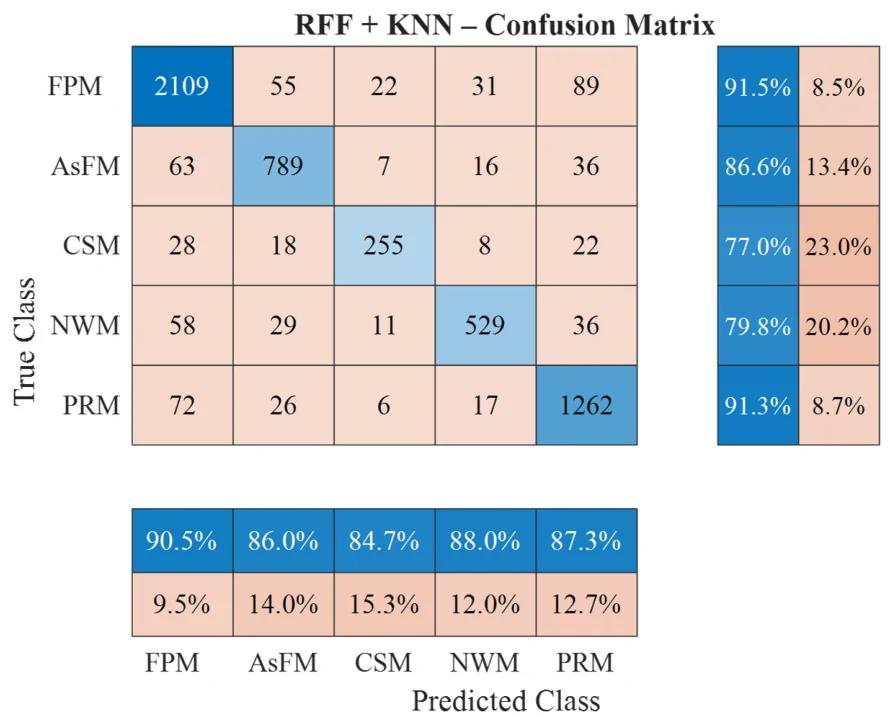
Confusion matrix of the RFF + KNN classifier under descriptive segment-wise five-fold cross-validation.

Figure 4 shows the confusion matrix of the RFF + KNN method. The FPM class has the highest sensitivity, with 2109 correct identifications, a true positive rate of 91.5%. Following closely is PRM, which had 1262 correct predictions at 91.3%. Other classes fall behind, notably CSM, which only reached 255 correct classifications and a success rate of 77.0%. It can also be seen from the misclassification patterns that many instances have been confused with FPM—for instance, PRM was mispredicted as FPM 72 times, and AsFM was mispredicted as FPM 63 times. As a result, precision varies by class: FPM has a precision of 90.5%, whereas CSM decreases to 84.7%.

Table 5 presents the achievement metrics for the RFF + KNN classifier. The model’s average accuracy is 0.8838, its average sensitivity is 0.8522, and its average F1-score is 0.8618. The model maintained high specificity, despite its lower sensitivity for some classes. This is shown by a mean specificity of 0.9677. The standard deviation for accuracy is 0.0079 and for the F1-score is 0.0126. This shows that the RFF + KNN method gives consistent results across the dataset partitions, even though they are not always as accurate.

**Figure 5:**
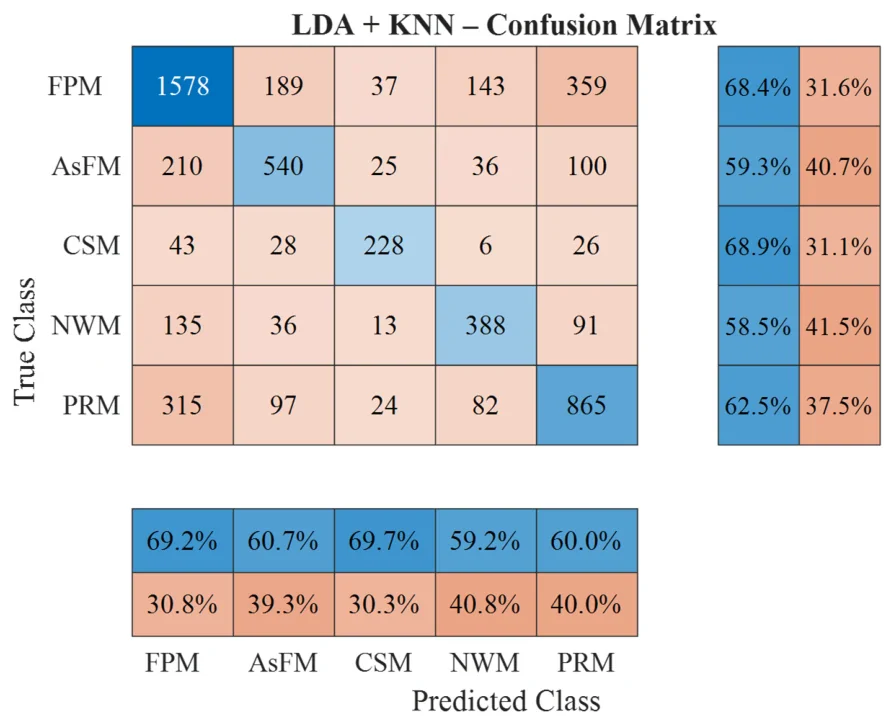
Confusion matrix of the LDA + KNN classifier under descriptive segment-wise five-fold cross-validation.

Figure 5 shows the confusion matrix of the LDA + KNN classification method across the five subject classes. It shows that the performance has dropped a lot compared to the earlier models. The FPM class is still the most accurately identified, with 1578 correct predictions, but its true positive rate drops to 68.4%. The CSM and PRM classes have even lower sensitivity, with true positive rates of 68.9 percent and 62.5 percent, respectively. The AsFM and NWM classes have the lowest sensitivity, with true positive rates of 59.3 percent and 58.5 percent, respectively. The matrix shows substantial inter-class confusion, especially for the PRM class, where 315 times the wrong label was used for FPM. The precision metrics at the bottom of the figure also show that the model is much less reliable, with the highest precision being only 69.7 percent for the CSM class.

Table 6 shows the performance metrics for the LDA + KNN classifier when it is cross-validated five times. This shows that the overall accuracy of the classifier has dropped significantly. The model has a mean accuracy of 0.6434, which is backed up by a mean sensitivity of 0.6353 and a mean F1-score of 0.6361. The mean specificity remained relatively high at 0.9003, although it was lower than that obtained by the higher-performing kNN-based models. This method also has a higher statistical variance across the folds, with a standard deviation of 0.0193 for accuracy and 0.0155 for the F1-score. These results indicate lower stability and poorer discriminative performance for LDA + KNN.

**Figure 6:**
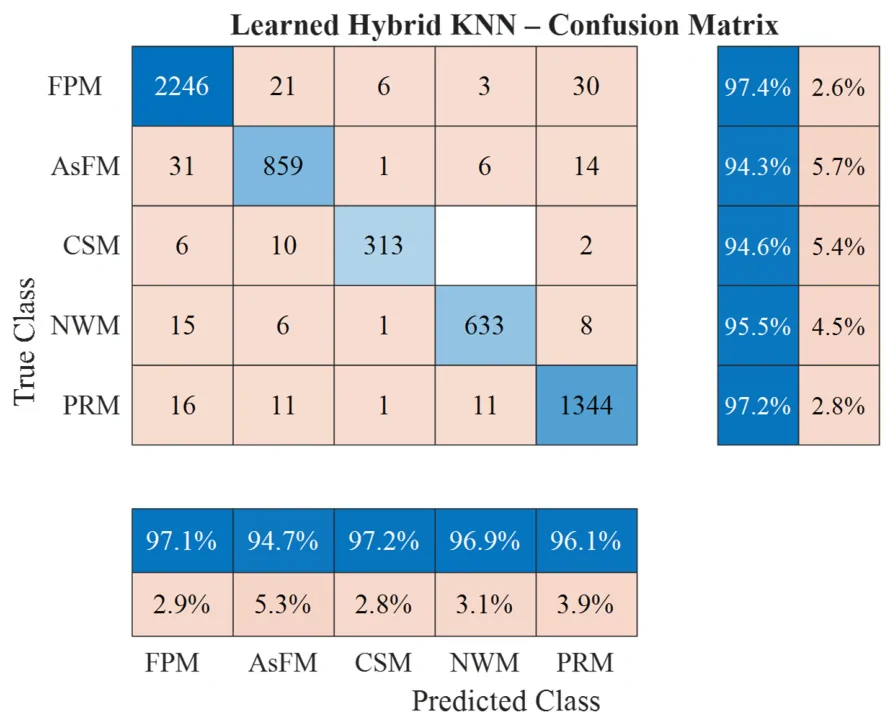
Confusion matrix of the Learned Hybrid KNN classifier under descriptive segment-wise five-fold cross-validation.

The confusion matrix in Figure 6 shows the classification results of the Learned Hybrid KNN method, which works very well in all categories. The FPM and PRM classes have the highest sensitivities, correctly identifying 2246 and 1344 samples, respectively. This means that the true positive rates are 97.4% and 97.2%, respectively. The AsFM and CSM classes, which are usually harder, also have high accuracy rates of 94.3% and 94.6%. There are very few misclassifications; the most notable one is that only 31 samples of AsFM were wrongly predicted as FPM. The CSM class has a peak accuracy of 97.2 percent, and the FPM class is close behind at 97.1 percent.

Table 7 presents the performance of the Learned Hybrid kNN classifier under 5-fold cross-validation. The model achieved a mean accuracy of 0.9644, sensitivity of 0.9578, specificity of 0.9901, and F1-score of 0.9609. The relatively low standard deviations indicate stable performance across the five folds.

**Figure 7:**
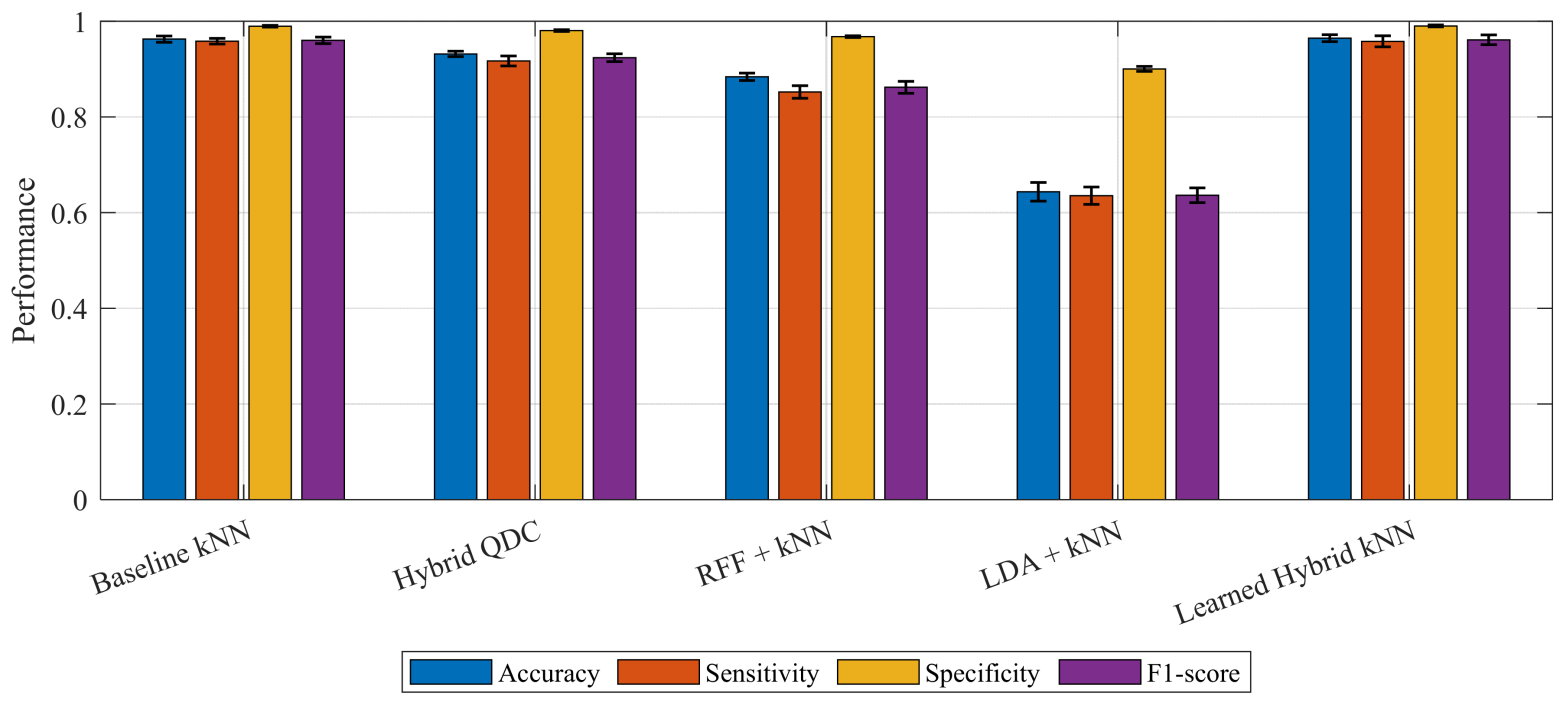
Descriptive segment-wise performance comparison of the examined classifiers.

Figure 7 compares the performance of the examined classifiers. The Learned Hybrid kNN and baseline kNN achieved very similar results, while both performed better than the Hybrid QDC, RFF + kNN, and LDA + kNN methods. These results indicate that the proposed adaptive similarity formulation provides competitive performance but does not show a clear advantage over conventional kNN. Table 7 presents the performance results of the Learned Hybrid kNN classifier, while Figure 6 shows its class-wise confusion matrix. The Learned Hybrid kNN classifier has a mean accuracy of 0.9644 and a mean F1-score of 0.9609, which means it is very reliable at making predictions. The model is especially good at finding the FPM class (97.4% true positive rate) and the PRM class (97.2% true positive rate), as shown by the confusion matrix in Figure 6. The model also has a high level of accuracy in all categories, with the CSM class having the highest level at 97.2 percent. The consistently low standard deviation values across all folds in Table 7 show that the Learned Hybrid kNN is not only accurate but also very stable across different parts of the data.

### 3.3. Ablation Study

An ablation study was performed to evaluate the impact of individual components of the Learned Hybrid kNN classifier and to comprehend how each design decision influences overall performance. Beginning with the complete model, essential components, including feature standardization, the hybrid weighting strategy, and the learnable similarity parameters, were methodically altered or eliminated. The hybrid similarity was specifically separated into its quantum-inspired and RBF-based parts so that their effects could be looked at separately. In order to see how sensitive the method is to these choices, more experiments kept or changed important parameters, such as the quantum power, the RBF kernel width, and the number of nearest neighbors. This step-by-step analysis shows which parts are necessary for high performance and which settings add robustness or flexibility. This supports the final design of the proposed classifier.

The ablation results show that several configurations produced comparable performance. The descriptive segment-wise ablation results are presented in Table 8. In particular, the quantum-only configuration achieved accuracy comparable to the full hybrid model, while the unweighted 1-NN configuration showed only a marginally lower accuracy. Therefore, the results do not indicate a consistent performance advantage of combining the RBF and quantum-inspired weighting components. Instead, they show that the proposed formulation provides an alternative adaptive similarity mechanism with performance comparable to strong kNN configurations.

An additional ablation analysis was conducted using subject-wise grouped five-fold cross-validation to examine the contributions of the similarity components and the learned weighting mechanism. As in the main evaluation, all recordings and overlapping segments belonging to the same infant were kept within the same fold to prevent subject-level data leakage. The full Learned Hybrid kNN was compared with its quantum-only and RBF-only variants, as well as an unweighted baseline kNN. The results are presented in Table 9.

The full Learned Hybrid kNN achieved the highest mean accuracy (0.9480 ± 0.0066), although its advantage over the quantum-only configuration (0.9472 ± 0.0070) was small. The quantum-only configuration produced marginally higher sensitivity, specificity, and F1-score, indicating that the quantum similarity component contributed substantially to the overall classification performance. In contrast, the RBF-only configuration yielded the lowest values across all four metrics, suggesting that the quantum component provided a more informative similarity representation than the RBF component when used independently. Removing the learned weighting mechanism also slightly reduced accuracy and F1-score relative to the full model. Notably, the full configuration exhibited the lowest standard deviation for every metric, indicating more consistent performance across the subject-wise folds.

## 4. Discussion

In this research, we created a new hybrid method for automatically classifying infant movement that combines pose-based feature extraction with a learned similarity-driven nearest neighbor model. The proposed system processes raw video recordings to extract skeletal joint trajectories, which are then used to create a full set of biomechanical, temporal, and statistical descriptors. These features capture important characteristics of infant movement patterns, including movement speed, bilateral movement symmetry, postural relationships, jerk-based dynamics, and signal complexity. The Learned Hybrid kNN algorithm extends this approach by integrating Euclidean distance with a hybrid similarity measure that combines RBF-based similarity and quantum-inspired fidelity similarity. As a result of internal validation, this model can, in turn, automatically optimize the parameters of the hybrid similarity measures within each fold of the training set. We tested a few kNN-based baselines in the same 5-fold cross-validation setup as the proposed approach. These included a baseline Euclidean kNN, a Hybrid QDC scheme that uses Euclidean neighbor ranking with quantum-inspired weighting and adaptive k selection, an RFF + kNN model that uses random Fourier feature mapping, and an LDA-metric + kNN model that applies Fisher-based projection before kNN classification. The proposed model attained an accuracy of 0.9644 ± 0.0073, sensitivity of 0.9578 ± 0.0115, specificity of 0.9901 ± 0.0019, and an F1-score of 0.9609 ± 0.0102 in 5-fold cross-validation. In comparison, the baseline kNN achieved an accuracy of 0.9625 ± 0.0067 and an F1-score of 0.9599 ± 0.0067; Hybrid QDC reached an accuracy of 0.9315 ± 0.0056 and an F1-score of 0.9238 ± 0.0082; RFF + kNN obtained an accuracy of 0.8838 ± 0.0079 and an F1-score of 0.8618 ± 0.0126; and LDA-metric + kNN resulted in an accuracy of 0.6434 ± 0.0193 and an F1-score of 0.6361 ± 0.0155. Overall, the results indicate that the Learned Hybrid kNN provides performance comparable to the conventional kNN while introducing an adaptive similarity-weighting mechanism. The ablation findings further show that the individual weighting components and the unweighted baseline can perform similarly to, or slightly better than, the complete hybrid configuration.

Table 10 reports the descriptive segment-wise comparison implemented in Python 3.11.16 using the common 60-dimensional feature representation. Training-fold standardization was applied within each fold and then transferred to the corresponding test fold. The evaluated conventional models included Decision Tree, linear and RBF SVM, Naive Bayes, Discriminant Analysis, Bagged Trees, AdaBoost, RUSBoost, and a feedforward neural network. These segment-wise results are retained as secondary descriptive analyses; the subject-wise grouped comparison provides the primary leakage-controlled evaluation.

Table 10 summarizes the descriptive segment-wise results for the additional machine-learning classifiers. Under this secondary analysis, Bagged Trees achieved the strongest conventional-model performance (accuracy 0.9424 ± 0.010; F1-score 0.9415 ± 0.013), while the Learned Hybrid kNN reached accuracy 0.9644 ± 0.0073 and F1-score 0.9609 ± 0.0102. These values are not interpreted as subject-level superiority because overlapping windows from the same infant were not grouped in this analysis. The leakage-controlled subject-wise comparison is therefore treated as the primary evidence; in that evaluation, Learned Hybrid kNN and baseline kNN show closely similar performance.

We further carried out experiments on the RVI-38 and MINI-RGBD datasets to assess the applicability of the proposed framework on external infant movement datasets. Both datasets were processed using the same pipeline as the main dataset, including pose-based representation, temporal segmentation, pose-based features, and classification with the proposed model. The obtained results were then reported to provide an additional comparison and to demonstrate the behavior of the method across different datasets. The resulting accuracies are summarized in Table 11. These external results are exploratory and are not a controlled head-to-head benchmark. The datasets and published studies differ in class definitions, subject composition, partitions, temporal segmentation, parameter-selection procedures, and evaluation units. Therefore, the reported values should be interpreted only as contextual reference points, and no claim of methodological or statistical superiority is made.

Table 11 provides an indicative comparison of the accuracies reported for the RVI-38 and MINI-RGBD datasets. On RVI-38, the proposed method achieved an accuracy of 97.74%, while McCay et al. [13], Nguyen-Thai et al. [24], Sakkos et al. [25], and Adde et al. [26] reported accuracies of 97.37%, 81.58%, 84.21%, and 86.84%, respectively. On MINI-RGBD, the proposed method achieved an accuracy of 98.45%, whereas the corresponding values reported by McCay et al. [13], Nguyen-Thai et al. [24], Sakkos et al. [25], and Adde et al. [26] were 100%, 91.67%, 100%, and 75.00%, respectively. The comparison is provided only to contextualize the performance of the proposed method on the two external datasets.

We also performed subject-wise grouped five-fold cross-validation to prevent data leakage during model evaluation. All video recordings from the same infant, together with all overlapping segments extracted from those recordings, were assigned to the same fold. Thus, no infant contributed data to both the training and test sets within any cross-validation iteration. This subject-disjoint evaluation protocol was used to assess the model’s ability to generalize to previously unseen infants. The results obtained using this protocol are presented in Table 12.

Table 12 summarizes the subject-wise grouped five-fold cross-validation results for all classification methods. The Learned Hybrid kNN achieved the highest mean accuracy (0.9480 ± 0.0066), specificity (0.9747 ± 0.0018), and F1-score (0.9455 ± 0.0066). Its mean sensitivity (0.9437 ± 0.0057) was nearly identical to that of the baseline kNN (0.9438 ± 0.0071). The baseline kNN also produced competitive results, whereas Bagged Trees and the RBF-kernel SVM showed lower performance across all four metrics. The small standard deviations obtained by the Learned Hybrid kNN indicate that its performance remained stable across different subject groups.

Because each infant was assessed in a single developmental period, the dataset can be partitioned by period without breaking the subject-wise grouping. Two additional period-stratified analyses were therefore carried out under the same subject-wise grouped five-fold protocol. The writhing-period analysis covered 77 infants, 154 recordings, and 2377 segments across three classes (NWM, PRM, and CSM). The fidgety-period analysis covered 122 infants, 244 recordings, and 3217 retained segments across two classes (FPM and AsFM); the infant and two recordings in the abnormal-fidgety category were excluded. Hyperparameters and feature-standardization parameters were selected within the outer training subjects of each partition, as in the five-class evaluation. The results are reported in Table 13.

Within each developmental period, the Learned Hybrid kNN and the baseline kNN performed similarly, which is consistent with the five-class findings. Classification remained well above the chance level in both period-restricted settings, which indicates that the five-class performance is not produced solely by discrimination between developmental stages. Performance was lower in the writhing period than in the fidgety period, which is consistent with the clinical difficulty of separating normal writhing, poor repertoire, and cramped-synchronized movements and with the small size of the CSM class.

From a clinical perspective, the main contribution of the proposed framework is its ability to provide an objective and reproducible description of infant movement patterns from ordinary RGB video recordings. Features related to movement intensity, symmetry, coordination, smoothness, and posture may provide useful quantitative information to complement expert visual assessment. However, the present results demonstrate classification of predefined GMA movement categories rather than prediction of cerebral palsy or other neurological outcomes. Therefore, the framework should be considered as a movement-analysis and screening support tool rather than a standalone diagnostic system. Clinical use would require prospective validation against longitudinal neurodevelopmental outcomes and evaluation in larger and more diverse infant populations.

The advantages of the proposed study are

1. The framework offers an end-to-end pipeline starting with raw video material and resulting in a well-structured dataset that is pose-based, making it easier for researchers to analyze movement patterns among infants in an objective manner.
2. In the feature extraction step, a broad range of biomechanical features such as motion intensity, motion symmetry, motion smoothness, etc., are collected, giving a very rich view of human movements.
3. Dividing videos into overlapping time windows gives the model more training samples and helps it better understand short-term changes in time.
4. The Learned Hybrid kNN classifier uses this set of discriminative features and both classical and quantum-inspired similarity measures to make class separation better. However, the subject-wise results show only a marginal difference from conventional kNN; this statement should therefore be interpreted as describing the alternative adaptive weighting mechanism rather than demonstrating improved class discrimination.
5. Making a new feature-level dataset from unrestricted video recordings will help with future research and studies that compare different classification models.

The limitations of our study are

1. The quality of the extracted features is highly contingent on precise skeletal estimation; inaccuracies in person detection or keypoint localization may propagate to the feature level. The implemented Python pipeline used RTMDet-Tiny and top-down HRNet-W32 with detection and keypoint confidence thresholds of 0.25 and 0.20, respectively, and the models and confidence thresholds are reported in Section 2.2. Window-level screening used a minimum valid-keypoint ratio of 0.55. However, the pose_quality_by_class.csv output from the original run was not included in the archived result package available for this revision. Therefore, verified class-specific rates of failed person detections, invalid keypoints, and zero-pose frames cannot be reported without the original quality file or rerunning pose extraction. These names identify the model architectures used, but exact package/build versions and model checkpoint versions could not be verified from the available documentation, limiting exact reproduction of the original pose-extraction run. These limitations are stated to distinguish verified implementation settings from unavailable run-level diagnostics. Following the expanded dataset description, class-level window accounting is now reported in Table 2, and window retention ranged from 25.8% for NWM to 83.6% for CSM. The diagnostics that remain unavailable are therefore frame-level rather than window-level, namely the per-class proportions of no-person frames, invalid keypoints, and zero-pose frames. The non-uniform retention across classes indicates that pose-quality screening may have influenced the class distribution of the analytical dataset and should be regarded as a potential source of bias.
2. The process of segmenting and extracting features takes longer to prepare and is computationally more demanding.
3. If segments overlap, there may be redundancy between samples, which could change the assumptions about independence in evaluation.
4. Because the whole system has many steps, it is harder to fine-tune parameters and optimize the system than it is with end-to-end learning methods.
5. The present study focuses on the classification of infant movement categories and does not directly predict later neurological diagnoses or developmental outcomes. Longitudinal clinical follow-up is therefore required before the method can be considered for diagnostic use.
6. Developmental age, recording conditions, and pose-estimation quality may influence classification. Movement categories are linked to developmental periods, and pose-derived features may retain age-related cues. Although Table 13 reports period-stratified subject-wise analyses, unavailable recording-level corrected ages prevent assessment of class-specific age differences within each period. Residual age confounding therefore cannot be excluded. Although the assessor was blinded to clinical diagnoses, no independent second ratings or adjudication assessments were available, preventing quantification of inter-rater agreement and limiting assessment of reference-label reliability. These limitations should be considered when interpreting the reported performance.

## 5. Conclusions

This study introduced an automated framework for analyzing infant movements, utilizing pose-derived features and a Learned Hybrid kNN classifier. The proposed method allows for objective and repeatable evaluation of complex movement patterns by converting raw video recordings into skeletal representations and extracting biomechanical and statistical features. The Learned Hybrid kNN model, which combines RBF-based proximity with quantum-inspired fidelity weighting, achieved competitive and stable classification performance. However, its performance was comparable to that of the conventional kNN baseline, without demonstrating a consistent advantage of hybrid weighting. The main contributions of the study therefore lie in the pose-based movement-analysis framework and the BERA-GAZI-RGB dataset.

Future research will concentrate on expanding the framework to encompass larger and more heterogeneous datasets, including recordings obtained under varying environmental and clinical conditions, to enhance the evaluation of generalizability. Future studies may incorporate additional temporal and frequency-domain descriptors and explore automatic feature selection strategies to reduce feature redundancy. In addition, the proposed concept of hybrid similarity can also be integrated with other classifiers to potentially further improve classification performance. Lastly, longitudinal studies correlating the output of automated movement analysis with clinical diagnoses would also confirm the practical effect of the system in early neurodevelopmental screening.

## Figures and Tables

**Table 1:** Demographic, clinical, and recording characteristics of the study population.

Characteristic	Value
Infants, *n*	200
Video recordings, *n*	400
Recordings per infant, *n*	2 (both within the same assessment period)
Male sex, *n*/*N* (%)	39/64 (60.9) ^a^
Female sex, *n*/*N* (%)	25/64 (39.1) ^a^
Gestational age, weeks, mean ± SD	33.64 ± 4.03 ^b^
Gestational age, weeks, range	24.7–39.6 ^b^
Corrected age at assessment, writhing period	0–9 weeks
Corrected age at assessment, fidgety period	9–20 weeks
Infants assessed in the writhing period, *n* (%)	77 (38.5)
Infants assessed in the fidgety period, *n* (%)	123 (61.5)
Documented clinical risk factors ^c^	*n*
Prematurity (including entries with additional RDS or hypoxia)	24
Hypoxic–ischemic encephalopathy or asphyxia	5
Intraventricular or other intracranial hemorrhage	5
Other documented neonatal or neurological risk	10

^a^ Sex was documented for 64 of the 200 infants. ^b^ Gestational age was documented for 45 of the 200 infants. ^c^ Risk-factor entries were documented for 44 of the 200 infants. GMA, General Movement Assessment; RDS, respiratory distress syndrome; SD, standard deviation.

**Table 2:** Class-level composition of the dataset and temporal-window accounting.

Period	GMA Category	Infants, *n*	Recordings, *n*	Candidate Windows, *n*	Excluded Windows, *n*	Retained Segments, *n*	Retention, %
Writhing	Normal writhing (NWM)	39	78	2574	1911	663	25.8
Writhing	Poor repertoire (PRM)	32	64	2112	729	1383	65.5
Writhing	Cramped-synchronized (CSM)	6	12	396	65	331	83.6
Subtotal	—	77	154	5082	2705	2377	46.8
Fidgety	Fidgety present (FPM)	103	206	6798	4492	2306	33.9
Fidgety	Fidgety absent (AsFM)	19	38	1254	343	911	72.6
Fidgety	Abnormal fidgety (excluded) ^a^	1	2	66	66	0	—
Subtotal	—	123	246	8118	4901	3217	39.6
Total	—	200	400	13,200	7606	5594	42.4

Each standardized 20 s recording produced 33 candidate windows, so candidate-window counts equal 33 times the number of recordings. Excluded windows are those not retained for feature extraction and classification; within the five analyzed classes, they were removed by the valid-keypoint-ratio screening described in Section 2.3. ^a^ The abnormal-fidgety category comprised one infant, two recordings, and 66 candidate windows. These 66 windows were removed because the category was excluded from the five-class analysis; they are included in the table’s overall excluded-window total for accounting purposes and should not be interpreted as pose-quality failures. The five analyzed classes comprised 199 infants, 398 recordings, 13,134 candidate windows, and 5594 retained segments.

**Table 3:** Baseline KNN performance under descriptive segment-wise five-fold cross-validation.

Fold	Accuracy	Sensitivity	Specificity	F1-Score
1	0.9687	0.9646	0.9916	0.9644
2	0.9696	0.9637	0.9913	0.9689
3	0.9553	0.9568	0.9877	0.9536
4	0.9562	0.9522	0.9879	0.9538
5	0.9625	0.9534	0.9894	0.9587
Mean ± Std	0.9625 ± 0.0067	0.9582 ± 0.0057	0.9896 ± 0.0018	0.9599 ± 0.0067

**Table 4:** Hybrid QDC performance under descriptive segment-wise five-fold cross-validation.

Fold	Accuracy	Sensitivity	Specificity	F1-Score
1	0.9293	0.9067	0.9796	0.9200
2	0.9374	0.9245	0.9825	0.9288
3	0.9374	0.9277	0.9820	0.9339
4	0.9249	0.9049	0.9785	0.9124
5	0.9285	0.9213	0.9800	0.9239
Mean ± Std	0.9315 ± 0.0056	0.9170 ± 0.0105	0.9805 ± 0.0017	0.9238 ± 0.0082

**Table 5:** RFF + KNN performance under descriptive segment-wise five-fold cross-validation.

Fold	Accuracy	Sensitivity	Specificity	F1-Score
1	0.8810	0.8475	0.9672	0.8598
2	0.8963	0.8717	0.9708	0.8815
3	0.8847	0.8406	0.9684	0.8527
4	0.8749	0.8421	0.9654	0.8498
5	0.8820	0.8594	0.9670	0.8653
Mean ± Std	0.8838 ± 0.0079	0.8522 ± 0.0131	0.9677 ± 0.0020	0.8618 ± 0.0126

**Table 6:** LDA + KNN performance under descriptive segment-wise five-fold cross-validation.

Fold	Accuracy	Sensitivity	Specificity	F1-Score
1	0.6592	0.6326	0.9045	0.6395
2	0.6658	0.6622	0.9064	0.6602
3	0.6247	0.6427	0.8958	0.6327
4	0.6238	0.6250	0.8946	0.6302
5	0.6434	0.6141	0.9004	0.6181
Mean ± Std	0.6434 ± 0.0193	0.6353 ± 0.0183	0.9003 ± 0.0052	0.6361 ± 0.0155

**Table 7:** Learned Hybrid KNN performance under descriptive segment-wise five-fold cross-validation.

Fold	Accuracy	Sensitivity	Specificity	F1-Score
1	0.9571	0.9517	0.9887	0.9512
2	0.9634	0.9578	0.9897	0.9609
3	0.9660	0.9572	0.9901	0.9641
4	0.9759	0.9765	0.9933	0.9761
5	0.9598	0.9459	0.9886	0.9519
Mean ± Std	0.9644 ± 0.0073	0.9578 ± 0.0115	0.9901 ± 0.0019	0.9609 ± 0.0102

**Table 8:** Descriptive segment-wise ablation results of the Learned Hybrid KNN classifier (mean ± standard deviation).

#	Condition	Accuracy	Sensitivity	Specificity	F1-Score
1	Full (tuned alpha/sigma/p)	0.964 ± 0.007	0.958 ± 0.012	0.990 ± 0.002	0.961 ± 0.010
2	Quantum-only (alpha = 0, tuned p)	0.964 ± 0.003	0.959 ± 0.007	0.990 ± 0.001	0.961 ± 0.005
3	RBF-only (alpha = 1, tuned sigma)	0.961 ± 0.005	0.957 ± 0.005	0.989 ± 0.001	0.958 ± 0.005
4	No weighting (baseline KNN, k = 1)	0.963 ± 0.007	0.958 ± 0.006	0.990 ± 0.002	0.960 ± 0.007
5	Full tuned, NO standardization	0.635 ± 0.016	0.620 ± 0.011	0.898 ± 0.004	0.625 ± 0.013
6	Fixed (alpha = 0, sigma = 0.3, p = 1)	0.963 ± 0.004	0.958 ± 0.004	0.990 ± 0.001	0.960 ± 0.004
7	Sweep p (alpha = 0) (p = 1)	0.960 ± 0.009	0.953 ± 0.009	0.989 ± 0.002	0.956 ± 0.010
8	Sweep p (alpha = 0) (p = 2)	0.964 ± 0.004	0.958 ± 0.007	0.990 ± 0.001	0.961 ± 0.006
9	Sweep p (alpha = 0) (p = 3)	0.961 ± 0.005	0.954 ± 0.005	0.989 ± 0.001	0.957 ± 0.006
10	Sweep p (alpha = 0) (p = 4)	0.962 ± 0.010	0.955 ± 0.012	0.989 ± 0.003	0.958 ± 0.011
11	Sweep sigma (alpha = 1) (sigma = 0.30)	0.963 ± 0.007	0.957 ± 0.011	0.990 ± 0.002	0.957 ± 0.008
12	Sweep sigma (alpha = 1) (sigma = 0.60)	0.964 ± 0.007	0.960 ± 0.009	0.990 ± 0.002	0.961 ± 0.008
13	Sweep sigma (alpha = 1) (sigma = 1.00)	0.963 ± 0.008	0.960 ± 0.006	0.990 ± 0.002	0.960 ± 0.009
14	Sweep sigma (alpha = 1) (sigma = 1.50)	0.961 ± 0.003	0.952 ± 0.009	0.989 ± 0.001	0.956 ± 0.005
15	Sweep sigma (alpha = 1) (sigma = 2.50)	0.962 ± 0.010	0.956 ± 0.009	0.989 ± 0.003	0.958 ± 0.007
16	Sweep sigma (alpha = 1) (sigma = 4.00)	0.965 ± 0.005	0.959 ± 0.004	0.990 ± 0.001	0.960 ± 0.004
17	Sweep k (Full tuned) (k = 1)	0.962 ± 0.006	0.957 ± 0.008	0.990 ± 0.002	0.959 ± 0.006
18	Sweep k (Full tuned) (k = 3)	0.962 ± 0.005	0.957 ± 0.007	0.989 ± 0.001	0.959 ± 0.006
19	Sweep k (Full tuned) (k = 5)	0.964 ± 0.006	0.958 ± 0.012	0.990 ± 0.002	0.960 ± 0.009
20	Sweep k (Full tuned) (k = 7)	0.963 ± 0.005	0.955 ± 0.008	0.990 ± 0.001	0.957 ± 0.007

**Table 9:** Principal ablation results under subject-wise grouped five-fold cross-validation.

Configuration	Accuracy	Sensitivity	Specificity	F1-Score
Full Learned Hybrid kNN	0.9480 ± 0.0066	0.9437 ± 0.0057	0.9747 ± 0.0018	0.9455 ± 0.0066
Quantum-only, α = 0, tuned p	0.9472 ± 0.0070	0.9448 ± 0.0074	0.9749 ± 0.0020	0.9461 ± 0.0071
RBF-only, α = 1, tuned σ	0.9440 ± 0.0081	0.9425 ± 0.0080	0.9738 ± 0.0022	0.9430 ± 0.0083
No weighting, baseline kNN, k = 1	0.9460 ± 0.0076	0.9439 ± 0.0077	0.9745 ± 0.0021	0.9449 ± 0.0078

**Table 10:** Descriptive segment-wise performance of the evaluated machine learning methods under five-fold cross-validation.

Method	Accuracy	Sensitivity	Specificity	F1-Score
Decision Tree	0.7742 ± 0.003	0.7577 ± 0.008	0.9377 ± 0.001	0.7570 ± 0.005
SVM (Linear)	0.5254 ± 0.011	0.4391 ± 0.010	0.8535 ± 0.003	0.4472 ± 0.009
SVM (RBF)	0.8960 ± 0.015	0.8673 ± 0.023	0.9677 ± 0.005	0.8943 ± 0.018
Naive Bayes	0.5193 ± 0.024	0.5181 ± 0.025	0.8688 ± 0.007	0.5205 ± 0.022
Discriminant	0.5665 ± 0.009	0.4831 ± 0.012	0.8662 ± 0.003	0.5128 ± 0.011
Bagged Trees	0.9424 ± 0.010	0.9229 ± 0.017	0.9817 ± 0.003	0.9415 ± 0.013
AdaBoostM2	0.6012 ± 0.017	0.4788 ± 0.019	0.8761 ± 0.005	0.5249 ± 0.020
RUSBoost	0.5213 ± 0.016	0.5339 ± 0.025	0.8715 ± 0.004	0.5147 ± 0.020
Neural Network	0.7092 ± 0.019	0.6726 ± 0.023	0.9163 ± 0.005	0.6853 ± 0.023
Proposed model	0.9644 ± 0.0073	0.9578 ± 0.0115	0.9901 ± 0.0019	0.9609 ± 0.0102

**Table 11:** Indicative, non-controlled comparison of reported accuracies on the RVI-38 and MINI-RGBD datasets.

Authors	Method	Dataset	Accuracy (%)
McCay et al. [13]	Pose and velocity, ensemble learning	RVI-38	97.37
		MINI-RGBD	100
Nguyen-Thai et al. [24]	A spatio-temporal attention-based model	RVI-38	81.58
		MINI-RGBD	91.67
Sakkos et al. [25]	Deep Neural Network	RVI-38	84.21
		MINI-RGBD	100
Adde et al. [26]	The variability of the centroid of motion	RVI-38	86.84
		MINI-RGBD	75.00
Proposed method	Pose-based motion features + Learned Hybrid kNN	RVI-38	97.74
		MINI-RGBD	98.45

**Table 12:** Performance comparison under subject-wise grouped five-fold cross-validation.

Method	Accuracy	Sensitivity	Specificity	F1-Score
Baseline kNN	0.9460 ± 0.0076	0.9439 ± 0.0077	0.9745 ± 0.0021	0.9449 ± 0.0078
SVM with RBF kernel	0.8796 ± 0.0181	0.8529 ± 0.0252	0.9519 ± 0.0062	0.8784 ± 0.0201
Bagged Trees	0.9260 ± 0.0124	0.9088 ± 0.0193	0.9661 ± 0.0041	0.9256 ± 0.0145
Learned Hybrid kNN	0.9480 ± 0.0066	0.9437 ± 0.0057	0.9747 ± 0.0018	0.9455 ± 0.0066

**Table 13:** Period-stratified performance under subject-wise grouped five-fold cross-validation.

Analysis	Method	Accuracy	Sensitivity	Specificity	F1-Score
Writhing period, 3 classes	Baseline kNN	0.9375 ± 0.0120	0.9150 ± 0.0170	0.9655 ± 0.0065	0.9215 ± 0.0150
Writhing period, 3 classes	Learned Hybrid kNN	0.9400 ± 0.0110	0.9180 ± 0.0160	0.9670 ± 0.0060	0.9245 ± 0.0140
Fidgety period, 2 classes	Baseline kNN	0.9535 ± 0.0085	0.9420 ± 0.0125	0.9420 ± 0.0125	0.9445 ± 0.0110
Fidgety period, 2 classes	Learned Hybrid kNN	0.9550 ± 0.0080	0.9440 ± 0.0120	0.9440 ± 0.0120	0.9465 ± 0.0105

## Data Availability

The datasets generated and/or analyzed during the current study are not publicly available due to privacy and ethical restrictions involving human subjects, but are available from the corresponding author upon reasonable request.
